# Quercetin Alleviates Oxidative Damage by Activating Nuclear Factor Erythroid 2-Related Factor 2 Signaling in Porcine Enterocytes

**DOI:** 10.3390/nu13020375

**Published:** 2021-01-26

**Authors:** Hai Jia, Yunchang Zhang, Xuemeng Si, Yuhang Jin, Da Jiang, Zhaolai Dai, Zhenlong Wu

**Affiliations:** 1 State Key Laboratory of Animal Nutrition, Department of Animal Nutrition and Feed Science, China Agricultural University, Beijing 100193, China; jiahai@cau.edu.cn (H.J.); B20173040318@cau.edu.cn (Y.Z.); sisi_sxm@cau.edu.cn (X.S.); SY20183040627@cau.edu.cn (Y.J.); B20193040341@cau.edu.cn (D.J.); daizhaolai@cau.edu.cn (Z.D.); 2 Beijing Advanced Innovation Center for Food Nutrition and Human Health, China Agricultural University, Beijing 100193, China

**Keywords:** quercetin, enterocytes, apoptosis, oxidative stress, Nrf2, glutathione

## Abstract

Oxidative stress has been implicated in the etiology of multiple gastrointestinal disorders, such as irritable bowel syndrome and inflammatory bowel disease. This study was conducted to evaluate effects of natural product quercetin on diquat-induced oxidative stress in porcine enterocytes and underlying mechanisms. Intestinal porcine epithelial cell line 1 (IPEC-1) cells pretreated with or without quercetin (5 μM, 24 h) were incubated with vehicle or diquat (100 μM) for 6 h. The results showed that diquat treatment induced apoptosis in a caspase-3-dependent manner, as accompanied by elevated reactive oxygen species (ROS) production, increased mitochondrial depolarization, and reduced the abundance of tight junction proteins. These adverse effects of diquat were remarkably abrogated by quercetin administration. Further study indicated that the protective effect of quercetin was associated with elevated protein abundance of nuclear factor erythroid 2-related factor 2 (Nrf2) and increased intracellular glutathione (GSH) content. Interestingly, the beneficial effects of quercetin on diquat-induced oxidative damage were abolished by all-trans-retinoic acid (Atra), a specific inhibitor of Nrf2, indicating a Nrf2-dependent regulation manner. The results show that quercetin attenuates diquat-induced cell injury by promoting protein abundance of Nrf2 and regulating GSH-related redox homeostasis in enterocytes. These findings provide new insights into a function role of quercetin in maintaining intestinal homeostasis.

## 1. Introduction

The intestinal epithelium acts as a barrier to prevent harmful intestinal contents and enable the selective uptake of ingested nutrients in intestine [1,2]. Intestinal epithelial cells are the most abundant cell type in the intestine and contributes to the mucosal barrier function with its intercellular tight junctions, which is essential for health of both humans and animals [2,3]. The dysfunction of intestinal epithelial barrier, increased gut permeability, enterocytes apoptosis can lead to intestinal injury, infections, diarrhea, and gastrointestinal disorders, as well as growth retardation and even death [4,5,6,7]. Oxidative stress has been considered as an important etiology of different human intestinal diseases, such as inflammatory bowel disease (IBD), peptic ulcers, celiac disease, and gastrointestinal cancers [8,9]. Excessive production of reactive oxygen species (ROS) is a major characteristic of oxidative stress as well as a contributive factor for intestinal mucosal damage [8,10]. Under normal circumstances, ROS production is maintained at a homeostatic level due to the endogenous antioxidant defense systems, such as superoxide dismutase (SOD), thioredoxin peroxidase (TPX), catalase (CAT), and glutathione peroxidase (GSH-Px) [11,12]. However, excessive production of ROS or a reduced antioxidant capacity leads to an accumulation of ROS in intestinal mucosa or enterocytes, thus causing oxidative stress and damage to macromolecules, including DNA damage [13,14], protein adduction [15], and lipid peroxidation [16,17]. In vivo and in vitro studies have shown that ROS can trigger apoptosis of intestinal epithelial cells and impair the intestinal mucosal barrier in animals [18,19].

Quercetin, a flavonoid abundantly found in fruits and vegetables, possesses multiple unique biological function including antioxidant, anti-inflammatory, anti-carcinogenic, and antiviral activities [20,21]. Growing evidence has indicated that quercetin has the therapeutic potential to prevent or treat various diseases, such as aging, cancer, diabetes, cardiovascular diseases, lung injury, and necrotizing enterocolitis (NEC) [22,23,24,25,26]. Administration of quercetin enhances the overall antioxidant capacity by regulating glutathione (GSH) synthesis in NEC patients [26]. Dietary supplementation of quercetin has been reported to improve antioxidant capacity and relieve the oxidative injury in the intestinal mucosa induced by Fusarium mycotoxins [11] or lipopolysaccharide [10]. In vitro studies have also proved that quercetin inhibits the production of ROS and prevents oxidative damage in endothelial cells and gastrointestinal epithelial cells [27,28,29,30]. However, the specific mechanisms responsible for the protection of quercetin are not well-elucidated.

Nuclear factor erythroid-derived 2-like 2 (Nrf2) is a key regulator that induces the transcription of antioxidant and cytoprotective genes under the stimulation of oxidative insults [31]. Previous studies have proved that Nrf2 regulates antioxidant defense system, therefore protecting against injury of liver, lung, or intestine [32,33]. In contrast, Nrf2-deficiency results in an increased sensitivity to oxidative insults in mice [34]. The balance of intestinal redox state is essential for heathy intestinal function, especially under pathological conditions. Growing evidence has shown that many plant bioactive substances can regulate Nrf2 expression, such as flavonoids [10,12], carotenoids [35], and polyunsaturated fatty acids (PUFA) [36,37,38]. Recent study has shown that *Eucommia ulmoides* flavones could promote Nrf2 activation, thus improving the antioxidant capacity and relieving intestinal oxidative damage in piglets [39]. Although quercetin has been used as component of traditional Chinese medicine for application in cardiovascular disease; however, its medicinal value for treatment of intestinal diseases remains to be further explored. In the present study, we hypothesis that quercetin has the potential to attenuate diquat-induced oxidative injury in porcine enterocytes by modulating the activities of Nrf2. This hypothesis was tested in a non-transformed newborn piglet jejunum epithelial cell line, which has been used as a useful in vitro model for nutrition related studies [18].

## 2. Materials and Methods

### 2.1. Reagents

Dulbecco’s modified Eagle’s F12 Ham medium (DMEM/F12) and fetal bovine serum (FBS) were obtained from Invitrogen (Carlsbad, CA, USA). Quercetin (#PHR1488) and diquat (#45422) were purchased from Sigma-Aldrich (St. Louis, MO, USA). Antibodies against PARP-1 (#9532), Caspase-3 (#9661S), and LC3A/B (#4108S) were obtained from Cell Signaling Technology (Beverly, MA, USA). Antibodies against Bcl-2 (sc-492), Bax (sc-493), and Nrf2 (sc-722) were obtained from Santa Cruz Biotechnology (Santa Cruz, CA, USA). Antibodies against Claudin-1 (#519000), Claudin-3 (#341700), Claudin-4 (#364800), Occludin (#404700), zonula occludens (ZO-1, #617300), ZO-2 (#389100), and ZO-3 (#364100) were obtained from Invitrogen (Carlsbad, CA, USA). Horseradish peroxidase (HRP)-labeled goat anti-rabbit IgG (#A0208), HRP-labeled goat anti-mouse IgG (#A0216), JC-1 and EdU detection kits were purchased from Beyotime Biotechnology (Haimen, China). GSH detection kit was obtained from Nanjing Jiancheng Bioengineering Institute (Nanjing, China). Unless indicated, all other chemicals were obtained from Sigma-Aldrich (St. Louis, MO, USA).

### 2.2. Cell Culture and Treatment

IPEC-1 cells were cultured in the completed medium of DMEM/F12 supplemented with 10% FBS and 1% penicillin-streptomycin mixture (100 U/L) in a humidified incubator under an atmosphere of 5% CO_2_ at 37 °C. IPEC-1 cells were seeded in different sizes of cell culture petri dishes or plates and adhered for 24 h. Next, cells were pretreated with quercetin (5 μM) or 0.05 % DMSO (served as control) for 24 h, and then challenged without (served as control) or with diquat (100 μM) for 6 h to establish a model of intestinal oxidative damage. The doses of quercetin were based on our cell viability assay and previous study as described [27]. For Nrf2 inhibition studies, IPEC-1 cells were pretreated with quercetin (5 μM) and/or all-trans-retinoic acid (Atra, 1 μM) for 24 h, and then challenged with diquat (100 μM) for 6 h. Cells were harvest and used to explore the underlying protective mechanism of quercetin on diquat-induced intestinal oxidative injury.

### 2.3. Cell Viability Assay

Cells (10,000/well) in 100 μL completed medium were seeded in a 96-well plate and adhered for 24 h. Next, cells were pretreated with 5 μM quercetin or 0.05 % DMSO (served as control) for 24 h, followed by diquat (100 μM) challenge for 6 h. After the end of treatment, the cells were incubated with CCK reagent (10 μL/well) purchased from Zoman Biotechnology (Beijing, China). The absorbance value is read at 450 nm and the cell viability was computed as a percentage relative to that of controls.

### 2.4. Cell Proliferation Assay

Cells seeded in a 24-well plate were treated as indicated and cell proliferations were measured as instructed by using a EdU cell proliferation kit with Alexa Fluor 594. Briefly, after incubation with 10 μM EdU for 2 h, cells were fixed, permeated, and labeled with Alexa Fluor 594 probes for 30 min in the dark. Nuclei were stained by incubation with DNA dye Hoechst 33,258 (1 μg/mL) for 5 min at 25 °C. The proliferating cells was visualized as red fluorescence under a fluorescence microscope (Zeiss Axiovert, Oberkochen, Germany). The proliferation rate was computed as a ratio of EdU to Hoechst 33,258 positive cells.

### 2.5. Apoptosis Analysis

Cells seeded in a 6-well plate were treated as indicated and apoptosis was assessed as instructed by a commercial apoptosis detection kit. Briefly, cells were harvested and washed with PBS twice. Next, cells were first incubated with annexin V- fluorescein isothiocyanate (FITC, 2.5 μg/mL) for 15 min in the dark, then with propidium iodide (PI, 50 μg/mL) for another 5 min at room temperature. Flow cytometer (Beckman Coulter, Miami, FL, USA) was used to analyze the cells. Data were acquired and evaluated by using CytExpert software. The proportion of apoptosis was computed as the number of apoptotic cells (Annexin-V-positive/PI-negative labelled early apoptotic cells and Annexin-V-positive/PI-positive labelled late apoptotic cells) to the total number of the cells.

### 2.6. Measurement of Mitochondrial Membrane Potential (MMP)

Cells seeded in a 6-well plate were treated as indicated and MMP of IPEC-1 cells was detected by a JC-1 assay kit (Beyotime Biotechnology, Haimen, China) according to the product’s instructions. Briefly, cells were harvested, resuspended, and stained by JC-1 for 20 min in 37 °C. The MMP and depolarization level of stained cells were analyzed by a fluorescence microscope (Zeiss Axiovert, Oberkochen, Germany) or a flow cytometer (Beckman Coulter, Miami, FL, USA).

### 2.7. Measurement of ROS

The intracellular ROS contents were detected by incubation with DCFH-DA (2′,7′-dichlorofluorescein diacetate) fluorescent probe as previously described [18]. Briefly, IPEC-1 cells were treated as indicated. After the end of treatment, cells were washed with PBS for three times and incubated with 10 μM DCFH-DA fluorescent probe in the serum-free DMEM/F12 medium for 20 min at 37 °C. The cells were washed with PBS and then analyzed via a fluorescence microscope (Zeiss Axiovert, Oberkochen, Germany) or a flow cytometer (Beckman Coulter, Miami, FL, USA).

### 2.8. Measurement of GSH

IPEC-1 cells were harvested via 0.25% trypsin-EDTA digestion and resuspended in cold PBS buffer. Next, cells were lysed by ultrasonic disruption and centrifuged at 3500 rpm for 10 min at 4 °C. The collected supernatants were analyzed for GSH level as instructed in a GSH detection kit (Nanjing Jiancheng Bioengineering Institute, Nanjing, China).

### 2.9. Immunofluorescence Staining

IPEC-1 cells fixed with 4% paraformaldehyde were permeabilized with 0.3% Trixon X-100. Next, cells were first incubated with a specific primary antibody against Nrf2 (1:200) overnight at 4 °C, and then incubated with cy3-labeled goat anti-rabbit IgG (1:500) for 1 h at 25 °C. Cell nuclei were stained by using DNA dye Hoechst 33,258 (1 μg/mL) for 5 min at 25 °C. Distribution of Nrf2 proteins in the nucleus and cytoplasm was visualized under a fluorescence microscope (Zeiss Axiovert, Oberkochen, Germany).

### 2.10. Western Blot Analysis

IPEC-1 cell proteins were extracted by rapid lysis of cells in RIPA buffer and protein concentration was measured by (bicinchoninic acid) BCA method. Equal amount of proteins (20 μg) were separated by 12% SDS-PAGE and transferred to PVDF membranes. The PVDF membranes were blocked and first incubated with a specific primary antibody (diluted at 1:2000) overnight at 4 °C, and then with a HRP-labeled secondary antibody (diluted at 1:2000) at 25 °C for 2 h. Protein bands were visualized by the Image Quant LAS 4000 mini system (GE Healthcare, Piscataway, NJ, USA). β-actin was used as an internal control.

### 2.11. Statistical Analysis

Statistical analysis was performed with the SPSS statistical software (Version 26.0). Data from three independent experiments are expressed as means ± SEM and evaluated by one-way ANOVA followed by the Duncan multiple comparison method. Means without a common letter are considered as a significant difference, *p* < 0.05.

## 3. Results

### 3.1. Quercetin Attenuated the Diquat-Induced Cell Viability Reduction in IPEC-1 Cells

**Figure 1:**
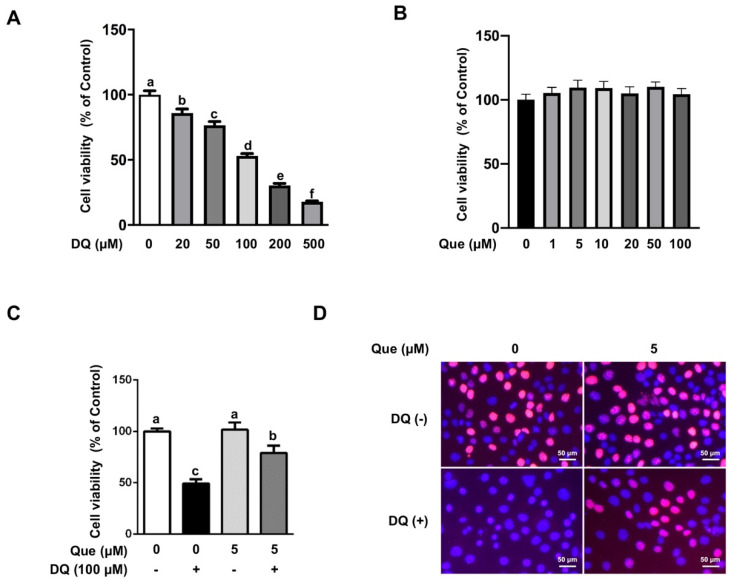
Effects of quercetin and/or diquat on cell viability in IPEC-1 cells. Cell viability of IPEC-1 pretreated with different dosages of (**A**) diquat for 6 h or (**B**) quercetin for 24 h were determined. IPEC-1 cells were pretreated with 5 μM quercetin for 24 h and then challenged with 100 μM diquat for 6 h. (**C**) Cell viability was measured by a CCK assay. (**D**) Cell proliferation was determined by EdU staining, magnification ×400. Representative experimental results were repeated in three independent experiments and values are means ± SEMs, *n* = 3. Means without a common letter are considered as a significant difference, *p* < 0.05.

Compared with the untreated cells, diquat treatment reduced cell viability in a dose-dependent manner (Figure 1A), and 100 μM of diquat reduced cell viability by almost 50%, which was used in the following study. Cell viability assay showed that treatment with quercetin (1–100 μM) for 24 h did not have cytotoxic effect on intestinal epithelial cells (Figure 1B). Based on this result and previous study [27], 5 μM of quercetin was applied in the following experiments to explore the protective effect and specific mechanism on diquat-induced oxidative damage. As shown in Figure 1C, diquat (100 μM) challenge led to a reduced cell viability (*p* < 0.05), and this effect was significantly attenuated (*p* < 0.05) by 5 μM of quercetin. Consistently, the numbers of proliferating cells were markedly reduced by diquat exposure, which was alleviated by quercetin (Figure 1D, Supplementary Materials Figure S1A). We also observed morphological alterations in diquat-treated cells, as shown by the appearance of nuclear condensation and cell boundary contraction, which were abolished by quercetin (Supplementary Materials Figure S1B). These results indicated that quercetin treatment attenuated diquat-induced viability reduction in IPEC-1 cells.

### 3.2. Quercetin Attenuated Diquat-Induced Apoptosis in IPEC-1 Cells

**Figure 2:**
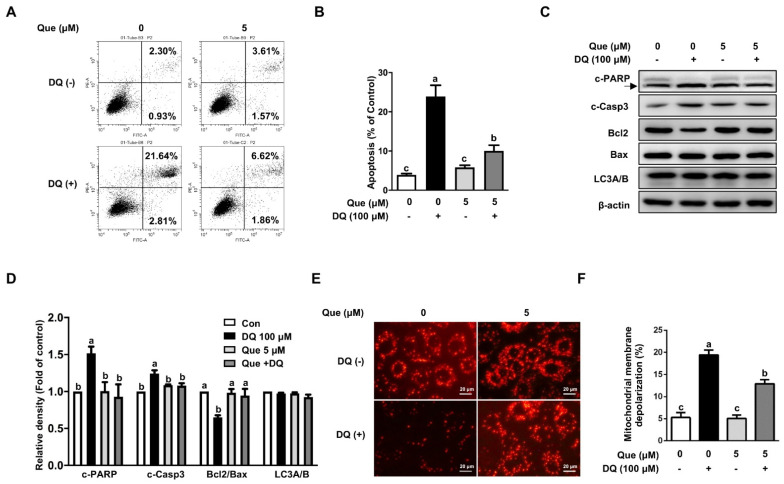
Quercetin attenuated diquat-induced apoptosis and mitochondrial membrane depolarization in IPEC-1 cells. IPEC-1 cells were pretreated with 5 μM quercetin for 24 h and then challenged with 100 μM diquat for 6 h. (**A**,**B**) Apoptosis were determined and analyzed by flow cytometry. (**C**,**D**) Protein abundances for c-PARP, c-Casp3, Bcl2, Bax, and LC3A/B were determined and quantified by Western blot assay. β-actin was used as the loading control. (**E**) Mitochondrial membrane potential was visualized by JC-1 staining in a fluorescence microscope (magnification ×400). (**F**) Mitochondrial membrane depolarization was analyzed by flow cytometry. Representative experimental results were repeated in three independent experiments and values are means ± SEMs, *n* = 3. Means without a common letter are considered as a significant difference, *p* < 0.05.

Compared with the control, diquat challenge led to greater apoptosis (23.9% vs. 4.7%), as evidenced by FCS analysis (Figure 2A,B).Apoptosis induced by diquat challenge was alleviated (*p* < 0.05) by quercetin supplementation, indicating an inhibitory effect of quercetin on apoptosis. In agreement with the phenotype observed, the protein abundances of cleaved-PARP (c-PARP) and cleaved-caspase-3 (c-Casp3), two characteristics of apoptosis, were markedly enhanced by diquat exposure, and this effect of diquat was attenuated by quercetin (Figure 2C,D). Furthermore, we investigate the mitochondrial depolarization level in diquat-induced cells. We found that diquat markedly decreased mitochondrial membrane potential by fluorescent staining, and this effect of diquat was alleviated by quercetin (Figure 2E). Consistently, cells challenged with diquat resulted (*p* < 0.05) in an increase proportion of mitochondrial depolarization by flow cytometry analysis, which was reversed (*p* < 0.05) by quercetin (Figure 2F, Supplementary Materials Figure S1C). Western blot analysis demonstrated that diquat treatment reduced (*p* < 0.05) the protein abundance of anti-apoptotic Bcl2 (Figure 2C,D), as compared with the control. Such adverse effects of diquat were markedly abolished (*p* < 0.05) by quercetin administration. In contrast, the protein abundance of pro-apoptotic Bax was not affected by diquat treatment (Figure 2C,D). Interestingly, diquat did not induce the protein abundance of LC3A/B, indicating an autophagy-independent cell death pathway in our intestinal epithelial cell injury model (Figure 2C,D). These results indicated that quercetin supplementation attenuated mitochondrial depolarization and apoptosis in diquat-challenged IPEC-1 cells.

### 3.3. Quercetin Attenuated Diquat-Induced ROS Accumulation and GSH Deprivation in IPEC-1 Cells

**Figure 3:**
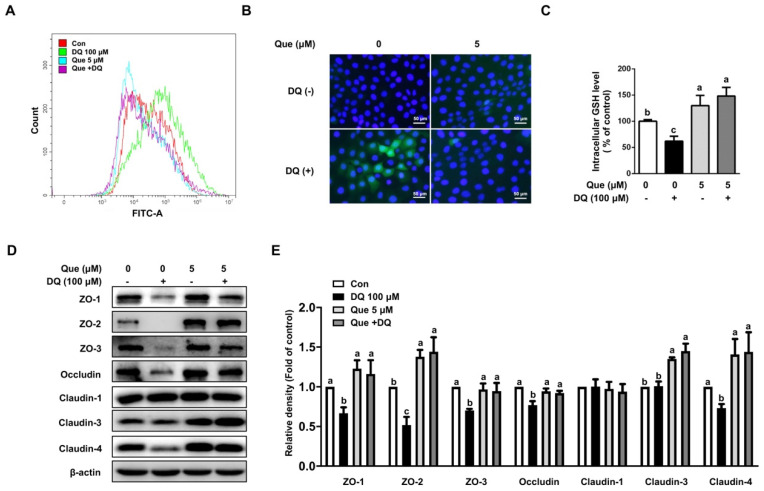
Quercetin attenuated diquat-induced ROS accumulation and reduced protein abundance of tight junction proteins in IPEC-1 cells. IPEC-1 cells were treated as described in Figure 2. (**A**) DCFH-DA-positive populations were detected by flow cytometry analysis. (**B**) Intracellular ROS levels were determined by fluorescence microscope (magnification ×200). (**C**) Intracellular GSH levels were measured. (**D**,**E**) Protein abundance for ZO-1, ZO-2, ZO-3, occludin, claudin-1, claudin-3, and claudin-4 were determined and analyzed by the Western blot assay. β-actin was used as the loading control. Representative experimental results were repeated in three independent experiments and values are means ± SEMs, *n* = 3. Means without a common letter are considered as a significant difference, *p* < 0.05.

Next, we explored the involvement of intracellular ROS levels in diquat-challenged cells and its contribution to cell death. As shown, diquat treatment led to the accumulation of ROS levels, as evidenced by a FITC-tagged peak shift observed at 6 h (Figure 3A). Consistently, immunofluorescence staining also showed that diquat treatment enhanced intracellular ROS levels, as compared with the control (Figure 3B). The addition of quercetin reduced the intracellular levels of ROS (Figure 3A,B). In addition, we also found that diquat challenge led to a decreased level of GSH, which was abrogated by quercetin (Figure 3C). These results indicated that quercetin improved the synthesis of GSH and eliminates intracellular ROS levels in diquat-induced IPEC-1 cells.

### 3.4. Quercetin Improved the Abundance of Tight Junction Proteins in Diquat-Induced IPEC-1 Cells

To assess the protective effect of quercetin on diquat-induced intestinal barrier injury, several classic tight junction proteins were evaluated. As shown, diquat challenge led to the downregulation of protein abundances of ZO-1, ZO-2, ZO-3, occludin, and claudin-4, as compared with the control, which were attenuated by quercetin co-treatment (Figure 3D,E). The protein abundance of claudin-3 was not affected by diquat challenge; however, the pretreatment of quercetin resulted in increased the protein abundance of claudin-3, regardless of diquat exposure (Figure 3D,E). Interestingly, diquat or quercetin treatment did not affect the protein abundance of claudin-1. These results indicated that quercetin alleviated the downregulation levels of tight junction proteins in diquat-induced IPEC-1 cells.

### 3.5. Quercetin Regulated the Nrf2 Signaling in IPEC-1 Cells

**Figure 4:**
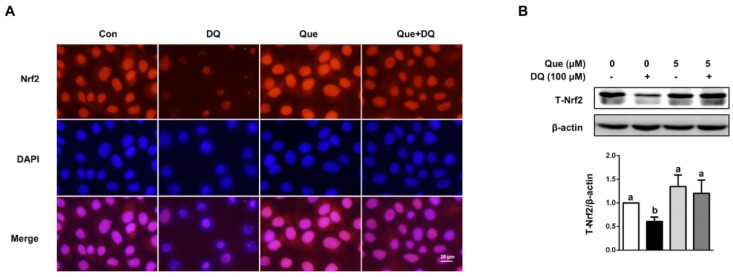
Quercetin attenuated diquat-induced the expression and distribution of Nrf2 in IPEC-1 cells. IPEC-1 cells were treated as described in Figure 2. (**A**) The distribution of Nrf2 in nucleus was determined by immunofluorescence staining (magnification ×400). (**B**) The abundance of Nrf2 in whole-cell proteins was determined and quantified by Western blot assay. β-actin was used as the loading control. Representative experimental results were repeated in three independent experiments and values are means ± SEMs, *n* = 3. Means without a common letter are considered as a significant difference, *p* < 0.05.

As observed in fluorescent staining results, diquat challenge reduced the protein distribution and abundance of Nrf2 in nucleus and cytoplasm, as evidenced by decreased red fluorescence intensity, which was reversed (*p* < 0.05) by quercetin (Figure 4A). In agreement with phenotype, diquat challenge led to decreased protein abundance of T-Nrf2 in IPEC-1 cells, as compared with the control, which was also attenuated (*p* < 0.05) by quercetin administration (Figure 4B). These observed results suggested that Nrf2 signaling pathway might be implicated in the protective function of quercetin in diquat-induced intestinal injury.

### 3.6. Quercetin Improved the Antioxidant Capacity of IPEC-1 Cells Challenged by Diquat in a Nrf2-Dependent Manner

**Figure 5:**
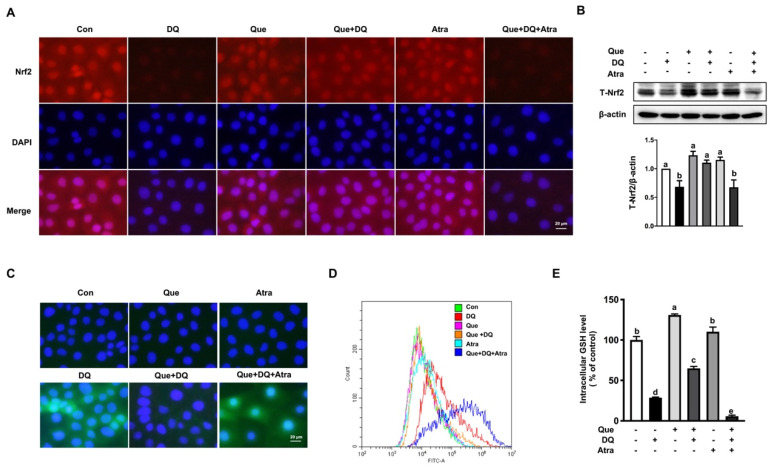
Quercetin protected against diquat-induced ROS production and GSH depletion in a Nrf2-dependent manner. IPEC-1 cells pretreated with or without quercetin (5 μM, 24 h) were challenged with diquat (100 μM) for 6 h in the presence or absence or Atra (1 μM). The distribution of Nrf2 in nucleus (**A**) and the abundance of T-Nrf2 (**B**) were determined. (**C**) Intracellular ROS levels were determined by fluorescence microscope (magnification ×400). (**D**) DCFH-DA-positive populations were detected by flow cytometry analysis. (**E**) Intracellular GSH levels were measured. Representative experimental results were repeated in three independent experiments and values are means ± SEMs, *n* = 3. Means without a common letter are considered as a significant difference, *p* < 0.05.

To explore the regulatory effect of Nrf2 on diquat-induced intestinal injury, cells pretreated with quercetin and/or Atra (1 μM) for 24 h, a Nrf2 inhibitor, were challenged with diquat (100 μM) for 6 h. As expected, diquat challenge led to decreased protein distribution and abundance of total Nrf2 and nucleus Nrf2, and these effects of diquat were attenuated by quercetin. Importantly, the protective effect of quercetin on the protein expression of Nrf2 under diquat challenge were markedly abrogated by Atra (Figure 5A,B). Diquat treatment led to morphological alterations, cell viability reduction, cell proliferation reduction, downregulated mRNA expression of *PCNA* (Supplementary Materials Figure S2A–E), which were prevented by quercetin supplementation. Moreover, the alleviated effect of quercetin on diquat-induced cell viability reduction was abolished by Atra (Supplementary Materials Figure S2A–S2E). We also found that quercetin regulated antioxidant capacity of IPEC-1 cells challenged by diquat in a Nrf2-dependent manner. As observed in flow cytometric analysis and immunofluorescence staining, the regulatory effect of quercetin on the diquat-induced ROS production were reversed by Atra (Figure 5C,D). Furthermore, the protective function of quercetin on diquat-induced GSH deprivation was abolished by Atra (Figure 5E), indicating that the Nrf2-regulated GSH redox system might be implicated in the regulatory effect of quercetin on diquat-induced intestinal epithelial cell damage.

### 3.7. Quercetin Protected Cells Against Diquat-Induced Apoptosis in a Nrf2-Dependent Manner

**Figure 6:**
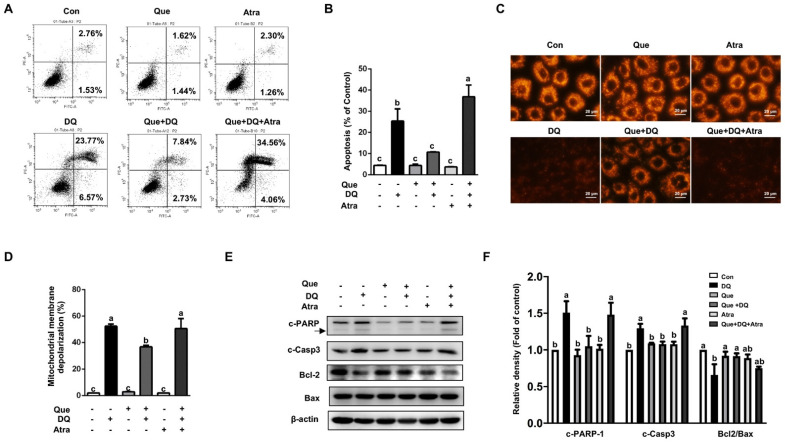
Quercetin protected against diquat-induced apoptosis in a Nrf2-dependent manner. IPEC-1 cells treated as described in Figure 5. (**A**,**B**) Cell apoptosis, (**C**,**D**) mitochondrial membrane depolarization, and (**E**,**F**) protein abundances of c-PARP, c-Casp3, Bcl2, and Bax were determined. Representative experimental results were repeated in three independent experiments and values are means ± SEMs, *n* = 3. Means without a common letter are considered as a significant difference, *p* < 0.05.

Compared with the control, cells challenged with diquat led to increased apoptosis, as evidenced by FCS analysis, which were reversed by quercetin (Figure 6A,B). Additionally, mitochondrial depolarization induced by diquat were reversed by quercetin (Figure 6C,D). Importantly, the regulatory effect of quercetin on diquat-induced apoptosis were abolished by Atra (Figure 6A–D). In addition, the protective function of quercetin on diquat-induced the upregulated protein abundances of c-PARP and c-Casp3, as well as the downregulated protein abundance of Bcl-2, were eliminated by Atra (Figure 6E,F). These results suggested that the protective effect of quercetin on diquat-induced apoptosis in intestinal epithelial cells might be attributed to the activation of Nrf2 signaling.

### 3.8. Quercetin Restored Tight Junction Proteins in Diquat-Treated Cells by Regulating Nrf2 Signaling

**Figure 7:**
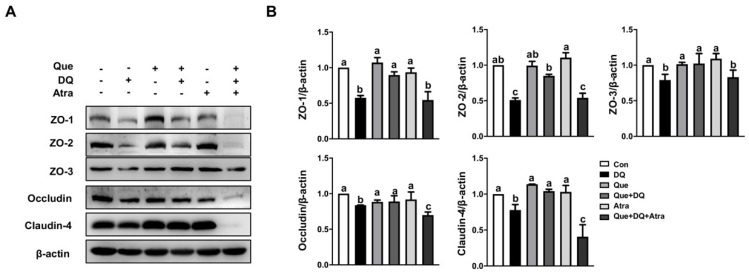
Quercetin regulated the abundance of tight junction proteins in a Nrf2-dependent manner. IPEC-1 cells were treated as described in Figure 5. (**A**,**B**) Protein abundance for ZO-1, ZO-2, ZO-3, occludin, and claudin-4 were assessed. Representative experimental results were repeated in three independent experiments and values are means ± SEMs, *n* = 3. Means without a common letter are considered as a significant difference, *p* < 0.05.

To validate the function role of Nrf2 signaling and its contribution to intestinal epithelial barrier injury, several classic tight junction proteins were evaluated by the application of Atra. As Western blot results shown, diquat challenge resulted in the reduced protein abundance of ZO-1, ZO-2, ZO-3, occludin, and claudin-4, and these effects of diquat were reversed by quercetin (Figure 7). Intriguingly, the protective function on quercetin on diquat-induced the downregulated protein abundance of tight junction was eliminated by Atra. These results indicated that Nrf2 signaling might contribute to the protective function of quercetin on tight junction proteins in diquat-challenge intestinal injury.

## 4. Discussion

In the present study, we investigated the protective function of quercetin against oxidative stress in an in vitro model of diquat-induced intestinal injury. Diquat induces oxidative stress and cell damage in porcine enterocytes, as demonstrated by increased ROS levels, decreased GSH content, impaired mitochondrial function, increased cell apoptosis, and decreased expressions of tight junction proteins. Interestingly, these adverse effects were greatly attenuated by quercetin. Further studies found that Nrf2 signaling pathway might participate in the protective effect of quercetin, as evidenced by the application of Atra, a specific inhibitor of Nrf2, which abolished the protective effect of quercetin. These findings highlight that the protective effect of quercetin is through Nrf2 signaling in neonatal intestinal health.

Intestinal mucosal barrier is critical to maintain the homeostasis of the human gastrointestinal tract [2]. ROS is an important challenge for intestinal health, causing oxidative stress and destroying intestinal barrier, thus leading to intestinal mucosal damage [8,10]. In this context, we hypothesis that quercetin has the potential to attenuate diquat-induced oxidative injury in porcine enterocytes. Diquat is a commercially bipyridyl herbicide and also a potent redox cycler, which transfers a free radical to molecular oxygen to form superoxide anions and then further produces other redox products [40]. It has reported that diquat leads to the redox system imbalance, destroys intestine barrier, and impairs mitochondrial function [41]. Previous research suggested that an ideal piglet model could be constructed by intraperitoneal injection of diquat, which is used to understand the effects of ROS generation and oxidative stress in intestine [42,43]. In this study, we found that diquat caused cell injury, as shown by reduced cell viability and proliferation, as well as morphological alterations. Diquat-induced intestinal epithelial cell injury has been proved to be associated with mitochondrial dysfunction in enterocytes [44]. As expected, we confirmed that diquat induced mitochondria-mediated apoptosis in intestinal epithelial cells, as demonstrated by activation of caspase-3 and PAPP-1. Mitochondria are sensitive to oxidative damage due to the lack of protection from histone and non-histone components [45]. We also noted that diquat resulted in mitochondrial membrane depolarization and induced the release of anti-apoptotic protein Bax from mitochondria and results in apoptosis. Quercetin is one of the flavonoids, which can be absorbed from food or supplements. Evidence has shown that plasma concentrations of quercetin are in the nanomolar level, which can reach to micromolar level, upon ingesting of quercetin-containing supplementation [46]. Previous studies also showed that 3–30 µM of quercetin had anti-inflammatory effects in the in vitro studies [47,48,49]. Moreover, 200 µM of quercetin increased anti-oxidative capacity in the intestinal epithelial cell [50]. Based on our cell viability results and previous reports, 5 µM of quercetin was used in our study to reveal a protective effect and its underlying mechanisms. In the present study, quercetin pretreatment increased mitochondrial membrane potential and decreased mitochondrial apoptotic pathway, thereby restoring the mitochondrial function in porcine enterocytes treated by diquat. These data indicated a protective effect of quercetin against cell death.

To further investigate the role of oxidative stress in diquat-induced apoptosis of intestinal epithelial cells, intracellular ROS level was measured via flow cytometry analysis and immunofluorescence staining. Accumulated ROS induced by diquat was remarkably eliminated by quercetin supplementation. As previous reports shown, quercetin is a flavonoid aglycone that has 3-OH groups and inhibits the formation of chain oxidation and terminates chain propagation in ROS production [51]. GSH-related redox homeostasis regulated by Nrf2 signaling is also an important contributes against oxidative injury in enterocytes [52]. As previous study shown, diquat was found to increase the oxidized glutathione (GSSG) content, decrease the GSH content, and increased the ratio of GSSG/GSH in the intestine of piglets [39], and similar results were also observed in our in vitro studies. In addition, diquat-induced GSH depletion was substantially reversed by quercetin supplementation. Quercetin has been proved to promote GSH synthesis and maintain GSH homeostasis in vivo and in vitro studies [26,53,54]. These results demonstrated that that quercetin could regulate GSH-related redox homeostasis and improve antioxidative capacity, and thus promote cell survival in enterocytes [27]. In addition, 3-OH groups in the flavonoid, such as quercetin, catechin, and myricetin, are potent inhibitors of lipid oxidation and act as hydrogen donor for the decomposition of hydrogen peroxide [51,55], which reduce or inhibit free radical toxicity and improve endogenous antioxidant capacity in the body, thus alleviate cell damage against diquat [51]. Therefore, quercetin treatment promotes the synthesis of GSH and helps to remove diquat-induced ROS production, thus protecting intestinal epithelial cell from free radical toxicity.

The intestinal epithelium acts as a protective barrier to prevent harmful materials from intestinal lumen, such as pathogens, and as the main site for the selective uptake of ingested nutrients in intestine, and thus is crucial for the intestinal homeostasis and host’s health [56]. The expression of tight junction proteins regulates the selective permeability between epithelial cells and maintains the intestinal epithelial barrier [57]. In vivo and in vitro experiments have proved that oxidative stress reduces the protein expression of tight junctions and destroys the intestinal barrier function in intestine epithelial cells [41,58,59]. In consistent with this, our in vitro study confirmed that diquat exposure decreased protein abundances of ZO-1, ZO-2, ZO-3, occludin, and claudin-4, indicating intestine barrier dysfunction in diquat-induced enterocytes. We also found that quercetin alone could not improve the above tight junction protein abundances, but it could alleviate diquat-induced tight junction protein expression reduction, indicating a protective role of quercetin against intestinal barrier injury.

In vivo studies have shown that Nrf2 regulates antioxidant defense system, therefore protecting against diquat-induced oxidative damage in intestine, liver, and lung, as observed in Nrf2^−/−^ mice [32,33]. As previous studies reported, activation of Nrf2 signaling pathways could improve antioxidant capacity, relieve oxidative stress and intestinal damage in piglets [39,60]. Our study explored the protective function of Nrf2 signaling on diquat-induced intestinal epithelial injury in vitro. As expected, we found that quercetin treatment improved the protein levels of Nrf2 in the nucleus fragment and enhanced intracellular content of GSH, thus restoring the intracellular GSH-related redox and promoting the survival of intestinal epithelial cells in response to diquat-induced oxidative insults. Importantly, these effects of quercetin were abrogated by a Nrf2 inhibitor, indicating a Nrf2-dependent regulation manner. In vivo study also showed that diquat injection decreased the activities of antioxidant enzymes SOD and GSH-Px in the jejunal mucosa [58], however, whether quercetin alleviates diquat-induced changes of antioxidant enzymes mediated by the Nrf2 signaling pathway, as reported in LPS-treated intestines of broiler chickens [10], remains to be further investigated in our model. In our studies, another novel finding is that the regulatory effect of quercetin on apoptosis, mitochondrial membrane potential, and tight junction proteins was abrogated by a Nrf2 inhibitor. The above results validated the contribution of Nrf2 signaling pathway on the protective effect of quercetin on cell survival and intestinal epithelial barrier function upon oxidative damage. Indeed, numerous in vitro and animal studies have been proved that natural product quercetin has a high medicinal value in preventing or treating multiple diseases, such as aging, cardiovascular diseases, necrotizing enterocolitis, and diabetes [22,23,24,25,26]. Our study confirmed that the key role of Nrf2 signaling in the protective effect of quercetin against diquat-induced oxidative injury in enterocytes. Multiple downstream targets, such as HO-1, NQO1, CAT, SOD, GSH-Px, and glutathione S-transferases (GSTs), have been reported as mediators for the anti-oxidative effects [61]. In our study, we observed the alterations of Nrf2 at protein level and cellular distribution, which can be regulated by quercetin. More studies are required to reveal which target is the main contributor for the beneficial effect of quercetin in diquat-challenged cells.

## 5. Conclusions

In conclusion, it was shown that natural product quercetin exerts protective effect in response to diquat-induced cell death and intestinal barrier dysfunction via Nrf2 signaling in enterocytes. Specifically, quercetin regulates intracellular redox states by scavenging ROS, enhancing intracellular GSH content, regulating tight junctions, and enhancing abundance of anti-apoptotic proteins in a Nrf2-dependent manner. This finding demonstrates great effects of quercetin on protection of porcine enterocyte in response to oxidative insults, which presents potential application in intestinal health care of human and animals.

## Data Availability

Data is contained within the article or supplementary material.

## Supplementary Materials

The following are available online at https://www.mdpi.com/2072-6643/13/2/375/s1, Figure S1: Proliferation, morphology, and mitochondrial membrane potential of IPEC-1 cells; Figure S2: Morphology, viability, proliferation, and mitochondrial membrane potential of IPEC-1 cells.

## Abbreviations

Atra, all-trans-retinoic acid; Bax, Bcl-2 associated X protein; Bcl-2, B-cell lymphoma 2; c-Casp3, cleaved-caspase-3; DAPI, 4′,6-diamidino-2-phenylindole; DQ, diquat; FITC, fluorescein isothiocyanate; GSH, glutathione; IPEC-1, intestinal porcine epithelial cell line 1; MMP, mitochondrial membrane potential; NEC, necrotizing enterocolitis; Nrf2, nuclear factor erythroid 2-related factor 2; PARP, poly ADP-ribose polymerase; Que, quercetin; ROS, reactive oxygen species; ZO, Zonula occluden.
