# Supplementary material for: Quercetin Alleviates Oxidative Damage by Activating Nuclear Factor Erythroid 2-Related Factor 2 Signaling in Porcine Enterocytes
Source: Nutrients. 2021 Jan 26;13(2):375. doi: 10.3390/nu13020375 (PMC7911945; doi:10.3390/nu13020375)
Supplement: Supplementary file 1 [file nutrients-13-00375-s001.pdf]

Supplementary Materials

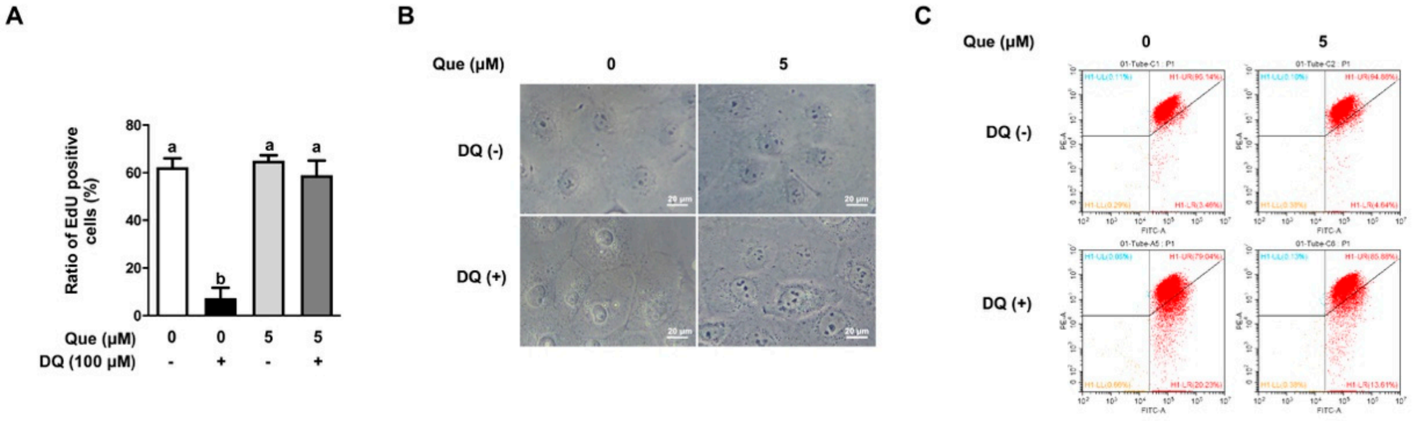

Figure S1. Proliferation, morphology, and mitochondrial membrane potential of IPEC-1 cells. IPEC-1 cells were pretreated with 5 μM quercetin for 24 h and then exposed to 100 μM diquat for 6 h. (A) Ratio of EdU positive cells was quantified and analyzed. (**B**) Cell morphology was observed by microscope, and images were taken under a microscope (magnification ×400). (**C**) Cells were stained by JC-1 and mitochondrial membrane potential were analyzed by flow cytometry. Representative experimental results were repeated in three independent experiments and values are means ± SEMs, n=3. Means without a common letter are considered as a significant difference , $p < 0.05$.

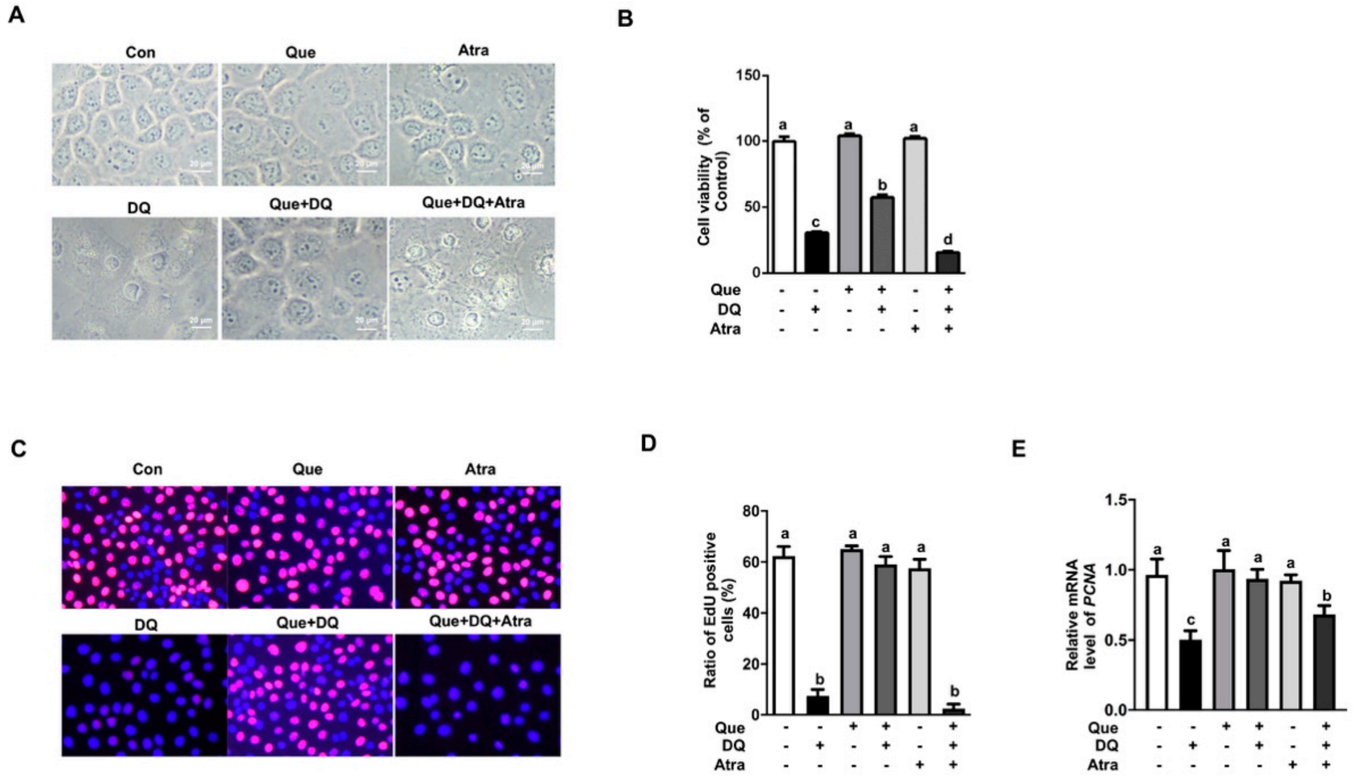

Figure S2. Morphology, viability, proliferation, and mitochondrial membrane potential of IPEC-1 cells. IPEC-1 cells pretreated with or without quercetin (5 μM, 24 h) were exposed to 100 μM diquat for 6 h in the presence or absence or Atra (1 μM). (**A**) Cell morphology, (**B**) cell viability, (**C**)

cell proliferation, (D) ratio of EdU positive cells and (**E**) mRNA level of *PCNA* were assessed. Representative experimental results were repeated in three independent experiments and values are means ± SEMs, $n=3$. Means without a common letter are considered as a significant difference , $p < 0.05$.
